# East Texas Medical Association

**Published:** 1887-06

**Authors:** J. E. Mayfield

**Affiliations:** Sec. E. T. M. A.; Nacogdoches, Tex.


### EAST TEXAS MEDICAL ASSOCIATION.

The third regular (quarterly) session of the E. T. M. A. was held at Timpson, Shelby Co., on Tuesday and Wednesday, April 5th and 6th 1887. The President, Dr. T. Y. T. Jameson, of the town of Rusk, presided, and Dr. A. H. McCord, of Cherokee, was chosen Secretary pro tem. Twenty odd members were in attendence, and fourteen new members were received, making total membership fifty. Much routine business transacted and social pleasures enjoyed—all hands delighted—a signal success.

Dr. A. M. Denman, of Lufkin, read an original paper on Placenta Prævia, and Dr. J. O. Lowrie, of Timpson, on Diseases of the Rectum. Both papers were creditable to the authors, were well received, and brought out interesting discussions.

Next meeting takes place at Lufkin, Tuesday, July 5th, 1887, 2 P. M., with following papers and discussions: Pathology of Inflammation, paper by Dr. J. T. Hoya, discussion by Drs. Bussey and W. G. Jameson. Typho-malarial Fever, paper by Dr. F. H. Tucker, discussion by Drs. Lacey and J. M. Abney. Pernicious Malarial Fever, paper by Dr. Treadwell, discussion by Drs. Watkins and S. A. Lowrie. Dysentery, paper by Dr. Horn, discussion by Drs. Wall and Barham. Uterine Hyperplasia, paper by Dr. McCord, discussion by Drs. Mayfield and J. O. Lowrie.

A committee was appointed to invite all physicians in east Texas to become members, or attend meetings and participate in discussions, and *Daniel's Texas Medical Journal* and the *Courier-Record* were by resolution requested, to publish such invitation, and also synopsis of proceedings of this session.

J. E. MAYFIELD, Sec. E. T. M. A.

P. S.—I am informed that delegates to the T. S. M. A. were not sent, because its laws make no provision for district associations to do so, and because the expenses would be too heavy, if delegates were admitted as from county association, or, at least, these laws are so vague as to leave serious doubts.

Also that a strong sentiment prevailed favoring the opening of the Medical Department of the State University at Galveston without delay. (The people voted it there. Who can change it.)

J. E. M.

NACOGDOCHES, TEX., April 26, 1887.

